# Online health information and public knowledge, attitudes, and behaviours regarding antibiotics in the UK: Multiple regression analysis of Wellcome Monitor and Eurobarometer Data

**DOI:** 10.1371/journal.pone.0204878

**Published:** 2018-10-24

**Authors:** Alistair Anderson

**Affiliations:** School of Geographical Sciences, University of Bristol, Bristol, United Kingdom; University of Campania, ITALY

## Abstract

**Background:**

Antimicrobial resistance is a global public health problem with some socially patterned drivers. The objective of the study was to investigate associations between use of and trust in the Internet as a source of health-related information and the public’s knowledge, attitudes, and behaviours regarding antibiotics.

**Methods:**

Two representative cross-sectional surveys (the 2015 Wellcome Monitor (n = 1524) and UK segment (n = 1330) of the 2016 Eurobarometer 85.1) covering knowledge about antibiotics and antibiotic consumption were analysed. Knowledge, attitude, and behaviour variables were analysed using regression in relation to demographic characteristics and use and trust in the Internet as a source of information.

**Results:**

The key findings of the analysis are that both use of the Internet as a source of medical research information (variable from the Wellcome Monitor) and trust in the Internet as a source of information about antibiotics (variable from the Eurobarometer) were independently and positively associated with knowledge, attitude, and behaviour regarding antibiotics. Additionally, knowledge about antibiotics was positively associated with behaviour with antibiotics (Wellcome Monitor) and attitude towards finishing antibiotic prescriptions (Eurobarometer). Higher levels of education were associated with better knowledge about antibiotics in both datasets. Older age was positively associated with behaviour and attitude regarding antibiotic consumption.

**Conclusions:**

The Internet is a resource for disseminating quality health information that has the potential to improve stewardship of antibiotics in the community. This study suggests that members of the UK public that use the Internet as a source of health-related information are more likely to be better informed about, and be more responsible with, antibiotics. This mode of information dissemination should be capitalised on to improve antimicrobial stewardship, and further research should examine what the most effective online information sources are in the UK and to what extent their association with behaviour is causal.

## Introduction

Antimicrobial resistance (AMR) is a global public health issue with both biological and social drivers. Educational interventions are often employed to improve public knowledge about antimicrobials and their appropriate use, with the aim of reducing socially patterned drivers such as inadherence to antibiotic courses or the pressuring of physicians by patients [1–7]. With health-related information becoming increasingly available to the public, the role of accessible resources of variable quality—such as the Internet—needs to be assessed with regard to the dissemination of information about antibiotics and their appropriate use.

In the UK there is a high but often superficial awareness of AMR, with the Wellcome Monitor [8] reporting that 91% of respondents had come across the term “antibiotic resistance” (though it was most commonly believed to mean the body becoming resistant) and the Eurobarometer survey [9] reporting that 92% of UK respondents agree that “unnecessary use of antibiotics can lead to them becoming ineffective”. Internationally, it has been consistently found that significant proportions of populations hold erroneous beliefs about the function and efficacy of antibiotics such as the belief that antibiotics are useful for treating viral infections such as flu [3, 6, 8, 10–13]. Important for understanding the role and efficacy of educational interventions regarding antibiotics is analysis of the association between knowledge regarding antibiotics, and attitudes and behaviours regarding antibiotics. One aspect of this association is the information sources used by the public for health-related information. The Internet is provides a variety of sources with a high and growing prevalence of use across a range of health scenarios, with studies in varied health contexts finding that the offline behaviour of consumers of online health information has been changed as a result of accessing online health information [14–17]. In Great Britain the Office for National Statistics [18] reports that 54% of adults used the Internet for health-related information in a three-month period in 2018, an increase of 30% since 2008. One example of the Internet being used to disseminate information about antibiotics is through social media, with previous studies finding that Twitter has experienced spikes in activity relating to antibiotics after national interventions and news announcements [19–20]. This suggests that the Internet has significant potential as a communication tool in this health context. A recent study in an Italian region found that the Internet is being used by the public to address a lack of knowledge regarding antibiotic use, however these Internet users were suggested as being more likely to self-medicate with antibiotics [21]. The authors of the Italian study argue that the key problem is not a lack of online information, but the quality of the information being consumed. This is a concern shared across health topics [22–27].

In the context of increasing use of the internet for health-related information and the imperative for good antimicrobial stewardship, the aim of this study was to assess whether the Internet as a source of health-related information is independently associated with good or poor knowledge levels about antibiotics, knowledge about antibiotic resistance, and attitudes and behaviours regarding the consumption of antibiotics in the UK.

## Methods

### Data sources

The data for this study were drawn from the Wellcome Trust Monitor Wave 3 (herein the ‘Monitor’) conducted by Ipsos Mori in 2015 [28], and Eurobarometer 85.1 [29] conducted by TNS Opinion for the European Commission in 2016. These representative surveys collected data on multiple areas relevant to AMR, including knowledge, attitude, and behaviour regarding antibiotics, as well as data on use of or trust in the Internet as a source of information.

The Monitor tracks changes over time in public views on science and biomedical research. The Monitor used a stratified sample in a random probability methodology to generate a representative sample of UK adults living in private residential accommodation, who were then surveyed using face-to-face interviews between 2^nd^ June and 1^st^ November 2015 [8]. The completed Monitor sample includes 1,524 cases.

The Eurobarometer series of surveys was established in 1974 to monitor the evolution of public opinion in EU Member States. Eurobarometer 85.1 [29] used a stratified sample in a random probability methodology to sample approximately 1000 resident individuals aged 15 or over in each EU Member State, who were surveyed using face-to-face interviews between 9^th^ April 2016 and 18^th^ April 2016. The completed UK segment of the Eurobarometer sample includes 1330 respondents.

The datasets differed on the weighting procedures used. The Monitor data was weighted to adjust for selection probabilities, non-response, and calibration with UK population totals by age, sex, and region [30]. The Eurobarometer supplied non-response weights based on age, sex, and regional characteristics [31]. Design weights were not made available for the Eurobarometer data.

### Statistical analysis

Statistical analysis was carried out using RStudio version 1.1.383.

Multiple regression analysis was performed using binary logistic and ordinal logistic regression to investigate which independent variables influenced Internet use and trust, knowledge about antibiotics, knowledge about antibiotic resistance, and behaviour/attitudes regarding antibiotic use. The construction of the dependent variables is detailed below, with knowledge variables being ordered categorical variables, and behaviour/attitude variables being binary. All models included the socio-demographic characteristics of age (15/18-31, 32–45, 46–62, 63+), sex (0 = male, 1 = female), education (in Monitor data: No qualifications, Level 1, GCSE, A-Level, Other Higher, First Degree, Postgraduate Degree; in Eurobarometer: Left education aged 15–16, 17–18, 19–21, and 22+), and employment type (not employed, self-employed, employed). In the UK, GCSE and A-Level qualifications are secondary level educational qualifications with GCSEs generally being undertaken when aged 15–16, and A-Levels generally being undertaken between ages 17–18. Compulsory academic education ends at 16. Whether or not the respondent had been prescribed antibiotics in the past year (0 = no, 1 = yes) was included in all models except the Monitor behaviour model.

In the models using Monitor data, the information source variables were whether the respondent reported using a hospital or doctor as a source of medical research information (0 = no, 1 = yes), and whether the respondent reported using at least one online source to seek medical research information (0 = no, 1 = yes) including among the options NHS-run websites, search engines, and social media. In models using Eurobarometer data, the information source variables were whether the respondent reported trusting at least one type of healthcare professional as a source of information about antibiotics (0 = no, 1 = yes), and whether the respondent reported trusting at least one online source of information about antibiotics (0 = no, 1 = yes) including official health websites, other health-related websites, health-related blogs, or social media. The specification of the Internet variables is therefore of a set of potential sources with the common characteristic of requiring use of the Internet. Binary logistic regression was used to characterise the Internet variables in terms of age, sex, education, and employment variables in order to inform later conclusions drawn from models assessing antibiotic-related dependent variables.

Odds ratios (ORs) with 95% confidence intervals (CIs) were estimated. Statistical significance was determined by the CIs not including an OR of 1.

### Knowledge about antibiotics

The variables accounting for respondents’ knowledge about antibiotics were constructed differently in each dataset.

For the Wellcome Monitor sample, the antibiotic knowledge variable was derived from a question that asked interviewees which of a series of options could be treated effectively by antibiotics. The options were viral infections, fungal infections, bacterial infections, colds, flu, and allergic reactions. The variable was constructed by subtracting for each respondent the mentions of any options other than bacterial, and adding any mention of bacteria. Any respondent that correctly answered that antibiotics only effectively treat bacterial infections had a score of 1, and respondents that responded with other options had scores <1 depending on how many incorrect responses they provided. The lowest score was -5.

For the Eurobarometer sample, the antibiotic knowledge variable is derived from a set of four true/false statement questions. The statements were “Antibiotics kill viruses”, “Antibiotics are effective against colds and flu”, “Unnecessary use of antibiotics makes them become ineffective”, and “Taking antibiotics often has side-effects such as diarrhoea”. Respondents’ scores on this variable were constructed by summing correct responses, with a range from 0 (no correct responses) to 4.

These variables were used as dependent variables in ordinal regression models predicting characteristics of better knowledge levels regarding antibiotics, and as independent variables in logistic regression models predicting appropriate behaviour or attitudes regarding antibiotic use.

### Knowledge about antibiotic resistance

This variable was only constructed for the Monitor, as there was no equivalent question in the Eurobarometer. The variable was derived from an open question in the Monitor, which asked respondents to define the term ‘antibiotic resistance’. The ad verbatim responses for this question were coded into 27 items by Monitor researchers. Respondents’ scores on this variable were constructed by summing correct responses and subtracting incorrect responses. This created a range of scores from -3 to 3. Due to low numbers of respondents on the two lowest scores, the categories were condensed from 7 categories to four by combining -3 and -2, -1 and 0, and 1 and 2.

This variable was used as a dependent variable for an ordinal regression model predicting knowledge about antibiotic resistance, and as an independent variable in a logistic regression model predicting appropriate behaviour with antibiotics.

### Behaviour and attitudes regarding antibiotics

The variables accounting for behaviour and attitudes to antibiotics were constructed differently in the two datasets due to different questions being asked in the surveys.

The Monitor asked respondents questions about how they had behaved the last time they had taken antibiotics. The variable in this study was derived from a question that asked respondents what they did with their most recent antibiotic prescription. The variable was coded as a binary variable where 1 represented respondents that had been prescribed the antibiotics and had also taken the whole course (n = 1259). Respondents that were currently taking antibiotics (n = 9) or had stopped taking them due to an adverse reaction (n = 7) were excluded from the variable. Due to the low number of cases reporting not completing their course of antibiotics (n = 93), the behaviour score was constructed such that 0 represented respondents that had taken prescribed antibiotics inappropriately, had self-medicated, or had never taken antibiotics (n = 249).

The Eurobarometer only asked respondents who had taken antibiotics in the past year how they had acquired the antibiotics. This reduced the available sample to the 472 cases that had consumed antibiotics in the past year. Out of these cases, 138 had been administered by a medical practitioner, and 314 were from a medical prescription. A response variable based on this data would therefore present a large reduction in sample size compared to the other models’ response variables in this analysis. The Eurobarometer also asked respondents when they thought it was appropriate to cease taking a course of antibiotics. The binary variable for this study was derived from this attitude question, with respondents answering that one should cease taking a course of antibiotics once it has been completed being represented by 1.

These variables were used as dependent variables in models predicting appropriate behaviour or attitude.

## Results

### Key sample characteristics

The key characteristics of the two samples for this study are presented in Table 1. The education variables used were not directly comparable, as the Monitor collected data on highest educational qualifications, whilst the Eurobarometer collected data on the age at which respondents left education. A weighted t-test comparison of age in each sample (t = -6.08, p<0.001) suggested a significant difference between the datasets at a 95% level of confidence. The weighted quartiles for age in each sample were similar (Monitor: 25% = 32, 50% = 47, 75% = 63, Eurobarometer: 25% = 31, 50% = 45, 75% = 62), and the average of the quartile cut-offs (25% = 32, 50% = 46, 75% = 63) was used to create identical categories for age in each dataset. A weighted chi-square comparison of quartile membership (χ^2^ = 5.62, dF = 3, p = 0.132) did not reject the null hypothesis that there was no difference between the two datasets in this regard. A weighted chi-square comparison of sex (*a5^2^ = 0.04, dF = 1, p = 0.833) showed no significant difference between the two datasets. Furthermore, no significant difference was found between the numbers of respondents that reported having been prescribed antibiotics for in the past 12 months (*a5^2^ = 0.04, dF = 1, p = 0.849). A significant difference was demonstrated between the datasets on the employment variable (*a5^2^ = 384.73, dF = 2, p<0.001). In the Monitor sample, 13% of respondents reported using a hospital or doctor to actively seek medical research information, with 35% reporting using the Internet for this purpose. In the Eurobarometer 92% of respondents reported trust in healthcare professionals as a source of information about antibiotics, with 17% trusting the Internet for information about antibiotics.

**Table 1:** Characteristics of the data.

	Wellcome Monitor			Eurobarometer		
	Variable	n	%	Variable	n	%
**Age**	**18–31**	301	19.75	**15–31**	234	17.59
	**32–45**	332	21.78	**32–45**	281	21.13
	**46–62**	411	26.97	**46–62**	324	24.36
	**63+**	465	30.51	**63+**	491	36.92
**Sex**	**Male**	695	45.60	**Male**	632	47.52
	**Female**	829	54.40	**Female**	698	52.48
**Education**	**No Qualifications**	292	19.16	**Left Education 15–16**	636	47.82
	**Level 1**	98	6.43	**Left Education 17–18**	249	18.72
	**GCSE**	279	18.31	**Left Education 19–21**	161	12.11
	**A-Level**	234	15.35	**Left Education 22+**	196	14.74
	**Other Higher**	225	14.76	**Still Studying**	75	5.64
	**First Degree**	233	15.29			
	**Postgraduate**	150	9.84			
**Employment Status**	**Not Working**	106	6.96	**Not Working**	739	55.56
	**Self-Employed**	188	12.34	**Self-Employed**	89	6.69
	**Employed**	1216	79.79	**Employed**	502	37.74
**Recent Antibiotic Consumption**	**Prescribed Antibiotic in Past Year**	326	21.39	**Prescribed Antibiotics in Past Year**	314	23.61
**Information Sources**	**Use Hospital or Doctor for Seeking Medical Research Information**	194	12.73	**Trust Health Care Professionals for Information About Antibiotics**	1223	91.95
	**Use Internet for Seeking Medical Research Information**	531	34.84	**Trust the Internet for Information About Antibiotics**	230	17.29

### Characteristics of internet variables

To initially contextualise the Internet variables used in this study, they were each regressed against the key socio-demographic variables used in the antibiotic-focussed models—specifically, age, gender, education, and employment. The results are presented in Table 2.

**Table 2:** Logistic regression analysis of internet variables.

	Use of Internet				Trust in Internet		
	OR	2.5% CI	97.5% CI		OR	2.5% CI	97.5% CI
**32–45 (Male)**	1.14	0.42	3.11	**32–45 (Male)**	1.29	0.38	4.36
**46–62 (Male)**	1.15	0.43	3.06	**46–62 (Male)**	3.98*	1.18	13.62
**63+ (Male)**	2.14	0.76	5.99	**63+ (Male)**	1.29	0.26	5.95
**Female (18–31)**	1.29	0.83	2.02	**Female (18–31)**	1.26	0.74	2.16
**Level 1**	1.95*	1.08	3.50	**Left Education 17–18**	1.51	0.98	2.31
**GSCE**	2.12*	1.34	3.40	**Left Education 19–21**	2.21*	1.41	3.46
**A-Level**	3.67*	2.33	5.87	**Left Education 22+**	3.25*	2.15	4.93
**Other Higher**	4.52*	2.87	7.26	**Still Studying**	3.36*	1.76	6.45
**First Degree**	5.74*	3.67	9.18				
**Postgraduate**	9.08*	5.58	15.10				
**Self-Employed**	1.22	0.71	2.12	**Self-Employed**	0.78	0.42	1.40
**Employed**	0.99	0.62	1.59	**Employed**	0.95	0.65	1.39
**32–45 (Female)**	1.01	0.54	1.89	**32–45 (Female)**	1.26	0.61	2.64
**46–62 (Female)**	0.82	0.44	1.52	**46–62 (Female)**	0.61	0.29	1.29
**63+ (Female)**	0.48*	0.25	0.92	**63+ (Female)**	0.64	0.25	1.66

* Statistical significance based on 95% CIs.

Both the use of the Internet for medical research information and trust in the Internet as a source of information about antibiotics were positively and confidently associated with education levels. From the Monitor data, education effect sizes increased with each extra level of education from Level 1 through to Postgraduate (OR = 1.95, 95% CI = 1.08–3.50; OR = 2.12, 95% CI = 1.34–3.40; OR = 3.67, 95% CI = 2.33–5.87; OR = 4.52, 95% CI = 2.87–7.26; OR = 5.74, 95% CI = 3.67–9.18, OR = 9.08, 95% CI = 5.58–15.10). Based on the Eurobarometer data, respondents still in education presented the largest positive education association (OR = 3.36, 95% CI = 1.76–6.45). The respondents that left education between the ages of 19 and 21 (OR = 2.21, 95% CI = 1.41–3.46), and 22 years or older (OR = 3.25, 95% CI = 2.15–4.93), presented positive associations with trust in the Internet as a source of information about antibiotics with the older increment presenting a stronger association.

There was one age association presented in each model. From the Monitor data, 63+ year old females (OR = 0.48, 95% CI = 0.25–0.92) were suggested as being less likely to use the Internet to seek medical research information than the reference group of 18–31 year old females. In the Eurobarometer data, 46–62 year old males (OR = 3.98, 95% CI = 1.18–13.62) were suggested as being more likely to trust the Internet as a source of information. Both of these age associations presented wide confidence intervals, suggesting imprecise estimates.

### Wellcome monitor models

The Monitor data was used to predict both knowledge about the efficacy of antibiotics and the concept of antibiotic resistance using ordinal response variables. The Monitor data was also used to predict responsible behaviour with a binary response. The results of the knowledge models are presented in Table 3.

**Table 3:** Ordinal regression analysis of monitor knowledge variables.

	Antibiotic Efficacy Knowledge			Antibiotic Resistance Knowledge		
	OR	2.5% CI	97.5% CI	OR	2.5% CI	97.5% CI
**32–45 (Male)**	1.57	0.66	3.76	0.33*	0.12	0.92
**46–62 (Male)**	3.70*	1.50	9.15	0.91	0.33	2.54
**63+ (Male)**	2.04	0.77	5.43	0.65	0.20	2.03
**Female (18–31)**	1.76*	1.21	2.57	1.19	0.77	1.84
**Level 1**	1.13	0.70	1.81	1.45	0.82	2.54
**GSCE**	2.31*	1.62	3.31	1.51	0.98	2.35
**A-Level**	1.85*	1.28	2.67	0.91	0.57	1.45
**Other Higher**	2.75*	1.89	4.00	1.45	0.93	2.27
**First Degree**	3.09*	2.11	4.52	1.72*	1.10	2.69
**Postgraduate**	2.95*	1.93	4.52	2.00*	1.23	3.27
**Self-Employed**	1.00	0.61	1.65	1.38	0.76	2.55
**Employed**	0.96	0.62	1.46	1.22	0.73	2.09
**Prescribed Antibiotic in Past Year**	0.90	0.70	1.14	0.85	0.64	1.12
**Use Hospital or Doctor for Medical Research Information**	0.96	0.70	1.33	0.92	0.64	1.33
**Use Internet for Medical Research Information**	1.37*	1.08	1.74	1.32*	1.01	1.73
**32–45 (Female)**	0.97	0.56	1.66	1.59	0.84	2.98
**46–62 (Female)**	0.70	0.40	1.22	1.07	0.57	2.03
**63+ (Female)**	1.05	0.58	1.92	1.18	0.59	2.39

* Statistical significance based on 95% CIs.

The model predicting knowledge about antibiotics’ efficacy presented multiple significant independent sociodemographic associations, as well as an independent association for use of the Internet. Age and sex were interacted in the model, and there was a strong positive association presented between 46–62 year old males (OR = 3.70, 95% CI = 1.50–9.15) and knowledge regarding the efficacy of antibiotics, and a weaker positive association presented for 18–31 year old females (OR = 1.76, 95% CI = 1.21–2.57) compared to the equivalent age group of males. Every level of education from GCSE upwards was positively associated with better knowledge about antibiotics’ efficacy, though the effect size did not increase directly with each increment of education. The effect size for A-levels (OR = 1.85, 95% CI = 1.28–2.67) was lower than GCSE (OR = 2.31, 95% CI = 1.62–3.31), Other Higher (OR = 2.75, 95% CI = 1.89–4.00), and First Degree (OR = 3.09, 95% CI = 2.11–4.52). The effect size for postgraduate qualification (OR = 2.95, 95% CI = 1.93–4.52) was also smaller than First Degree, with wider confidence intervals. Use of the Internet to actively seek medical research information (OR = 1.37, 95% CI = 1.08–1.74) was positively associated with better knowledge regarding antibiotics’ efficacy, with a smaller effect size than the significant levels of education. Having been prescribed antibiotics in the previous year (OR = 0.90, 95% CI = 0.70–1.14) was not significantly associated with better knowledge about antibiotics’ efficacy.

The model predicting correct definitions of antibiotic resistance presented fewer significant associations. The 32–45 year old male group (OR = 0.33, 95% CI = 0.12–0.92) was imprecisely but significantly associated with worse knowledge about antibiotic resistance. First Degree (OR = 1.72, 95% CI = 1.10–2.69) and Postgraduate qualification (OR = 2.00, 95% CI = 1.23–3.27) were both positively associated with knowledge about antibiotic resistance. An independent positive association was presented between the use of the Internet for medical research information (OR = 1.32, 95% CI = 1.01–1.73) and knowledge about antibiotic resistance. The low confidence bound was close to an OR of 1, suggesting that this association may not be as pronounced as the association between Internet use for medical research information and knowledge about antibiotics’ efficacy. The effect size was also smaller than any of the significant independent educational predictors. Having been prescribed antibiotics in the previous year (OR = 0.85, 95% CI = 0.64–1.12) was not significantly associated with better knowledge about antibiotic resistance.

The model predicting responsible behaviour, with results presented in Table 4, demonstrated socio-demographic and knowledge associations. Age and sex were not interacted in this model in order to maintain comparability with the equivalent Eurobarometer model, in which these variables were not interacted due to a low number of cases presenting poor attitude (n = 151/1330). In the Wellcome behaviour model, the 46–62 (OR = 2.76, 95% CI = 1.73–4.46) and over 62 (OR = 1.65, 95% CI = 1.04–2.63) year old age groups both presented independent positive associations with responsible behaviour, with the higher of the two age groups demonstrating a smaller effect size. Positive independent associations were also presented for female sex (OR = 1.86, 95% CI = 1.35–2.58) and being employed (OR = 1.88, 95% CI = 1.08–3.16). Better knowledge about the efficacy of antibiotics (OR = 1.24, 95% CI = 1.10–1.39) presented a positive association with more responsible antibiotic consumption, with precise confidence intervals relative to other predictors in the model. Knowledge about antibiotic resistance (OR = 1.13, 95% CI = 0.97–1.32) was not confidently presented as an independent indicator of behaviour with antibiotics. The confidence bounds for this variable were also precise relative to other variables in the model with the low bound proximate to an OR of 1, suggesting that an association between correct knowledge about antibiotic resistance and appropriate behaviour with antibiotics cannot be entirely dismissed. A positive association was presented between use of the Internet as a source of medical research information (OR = 1.49, 95% CI = 1.03–2.18) and appropriate behaviour with antibiotics, with a low confidence bound proximate to an OR of 1. There was no significant independent association presented in the model between responsible behaviour and the use of health professionals (OR = 1.42, 95% CI = 0.80–2.67) as a source for medical research information.

**Table 4:** Logistic regression analysis of monitor behaviour variable.

	Antibiotic Behaviour		
	OR	2.5% CI	97.5% CI
**32–45**	1.43	0.93	2.23
**46–62**	2.76*	1.73	4.46
**63+**	1.65*	1.04	2.63
**Female**	1.86*	1.35	2.58
**Level 1**	1.34	0.66	2.82
**GSCE**	0.78	0.46	1.32
**A-Level**	1.22	0.69	2.14
**Other Higher**	1.55	0.84	2.92
**First Degree**	1.68	0.92	3.09
**Postgraduate**	1.51	0.78	2.99
**Self-Employed**	1.43	0.74	2.77
**Employed**	1.88*	1.08	3.16
**Knowledge about Antibiotics**	1.24*	1.10	1.39
**Knowledge about Antibiotic Resistance**	1.13	0.97	1.32
**Use Hospital or Doctor for Medical Research Information**	1.42	0.80	2.67
**Use Internet for Medical Research Information**	1.49*	1.03	2.18

* Statistical significance based on 95% CIs.

### Eurobarometer models

The Eurobarometer data was used to predict knowledge about antibiotics with an ordinal response, and appropriate attitude towards antibiotic consumption with a binary response.

The model predicting knowledge about antibiotics, with results shown in Table 5, presented positive associations with knowledge for both self-employment (OR = 1.73, 95% CI = 1.12–2.70) and employment (OR = 1.37, 95% CI = 1.05–1.79). Having been prescribed antibiotics in the previous year (OR = 1.21, 95% CI = 0.95–1.54) was not significantly associated with better knowledge. The only significant age and gender association was positive for the 63+ male group (OR = 2.60, 95% CI = 1.03–6.60) with wide confidence intervals. Respondents that left education between the ages of 17 and 18 (OR = 1.39, 95% CI = 1.05–1.86), and above the age of 22 (OR = 1.87, 95% CI = 1.35–2.60), were positively associated with knowledge about antibiotics. Trust in the Internet as a source of knowledge about antibiotics (OR = 1.66, 95% CI = 1.26–2.19) was positively associated with correct knowledge about antibiotics, whilst trust in health care professionals as a source of knowledge about antibiotics (OR = 0.61, 95% CI = 0.41–0.90) was negatively associated with correct knowledge about antibiotics.

**Table 5:** Ordinal regression analysis of eurobarometer knowledge variable.

	Antibiotic Knowledge		
	OR	2.5% CI	97.5% CI
**32–45 (Male)**	1.11	0.45	2.74
**46–62 (Male)**	1.80	0.73	4.42
**63+ (Male)**	2.60*	1.03	6.60
**Female (18–31)**	1.26	0.86	1.85
**Left Education 17–18**	1.39*	1.05	1.86
**Left Education 19–21**	1.28	0.93	1.78
**Left Education 22+**	1.87*	1.35	2.60
**Still Studying**	1.46	0.92	2.32
**Self-Employed**	1.73*	1.12	2.70
**Employed**	1.37*	1.05	1.79
**Prescribed Antibiotics in Past Year**	1.21	0.95	1.54
**Trust Health Care Professional**	0.61*	0.41	0.90
**Trust the Internet**	1.66*	1.26	2.19
**32–45 (Female)**	1.65	0.94	2.90
**46–62 (Female)**	1.66	0.95	2.90
**63+ (Female)**	1.28	0.74	2.24

* Statistical significance based on 95% CIs.

The model predicting better attitude towards antibiotic consumption, results shown in Table 6, presented socio-demographic and knowledge associations. Due to the low number of cases presenting poor attitudes (n = 151/1330), age and sex were not interacted in this model. Each age group presented a positive association with attitude (OR = 1.81, 95% CI = 1.10–2.99; OR = 2.17, 95% CI = 1.28–3.72; OR = 5.43, 95% CI = 2.87–10.47), with higher age groups showing larger effect sizes. Being employed (OR = 1.88, 95% CI = 1.16–3.03) and having been prescribed antibiotics in the past year (OR = 1.75, 95% CI = 1.11–2.84) were both independently positively associated with attitude. Better knowledge (OR = 2.17, 95% CI = 1.85–2.57) was also positively associated with attitude, with narrow confidence intervals compared to the other predictors in the model. There were no significant associations between education and attitude towards finishing a course of antibiotics (OR = 1.60, 95% CI = 0.93–2.80; OR = 1.10, 95% CI = 0.63–1.97; OR = 0.63, 95% CI = 0.37–1.07; OR = 1.98, 95% CI = 0.98–4.04). Trust in the Internet as a source of information about antibiotics (OR = 3.59, 95% CI = 1.98–6.95) presented a strong positive association with attitude with a wide confidence interval. Trust in healthcare professionals as a source of information (OR = 1.97, 95% CI = 0.99–3.76) also presented a positive association, however the low confidence bound narrowly included 1. This suggests that trust in the Internet is a more important characteristic than either levels of education or trust in healthcare professionals for indicating good attitudes towards antibiotic use.

**Table 6:** Logistic regression analysis of eurobarometer attitude variable.

	Attitude Towards Finishing Course		
	OR	2.5% CI	97.5% CI
**32–45**	1.81*	1.10	2.99
**46–62**	2.17*	1.28	3.72
**63+**	5.43*	2.87	10.47
**Female**	1.35	0.95	1.94
**Left Education 17–18**	1.60	0.93	2.80
**Left Education 19–21**	1.10	0.63	1.97
**Left Education 22+**	0.63	0.37	1.07
**Still Studying**	1.98	0.98	4.04
**Self-Employed**	0.58	0.30	1.12
**Employed**	1.88*	1.16	3.03
**Prescribed Antibiotics in Past Year**	1.75*	1.11	2.84
**Knowledge about Antibiotics**	2.17*	1.85	2.57
**Trust Health Care Professional**	1.97	0.99	3.76
**Trust Internet**	3.59*	1.98	6.95

* Statistical significance based on 95% CIs.

## Discussion

This study analysed two random-probability sample surveys in order to assess the association between the Internet as a source of health-related information and the public’s knowledge relating to antibiotics and antibiotic resistance, and behaviour and attitudes regarding antibiotic consumption.

Knowledge about antibiotics was positively and independently associated with both behaviour and attitude. This supports previous findings that higher levels of knowledge about antibiotics are associated with more responsible consumption of antibiotics [12, 32–34] and more appropriate attitudes towards consumption [34–35], whilst diverging from studies that suggest that better knowledge about antibiotics is associated with less responsible behaviour [35–37].

In both the Monitor and Eurobarometer analyses the Internet variables presented positive independent associations with knowledge about antibiotics. The consistency between datasets of these positive independent associations with knowledge about antibiotics suggests that the Internet may be a productive space through which such knowledge is being disseminated in the UK. Both Internet variables also presented positive independent associations with respective behaviour and attitude variables. These findings are particularly significant considering the lack of positive independent associations presented by healthcare professional information variables. Furthermore, the consistency of the confident associations between behaviour/attitude and Internet variables in the absence of confident education associations suggests that preferred sources of information may be more important than level of education for the formation of attitudes and behaviours regarding antibiotic use.

This study suggests that, independent of key characteristics such as education level and age, members of the UK public that use online sources of health-related information are more likely to be better informed about antibiotics and use them more appropriately than those that do not make use of online sources of health-related information. This diverges from a recent Italian study [21] of Internet use for antibiotic-related information seeking that reported users of the Internet for health-related information as less informed, suggesting geographic differences in the association between Internet use and antibiotic-related knowledge and behaviour. Whilst the publics in these countries may be using the Internet to supplement information from healthcare professionals [21, 38], differences in online information provision by key stakeholders such as national health services may produce different outcomes in different geographic settings with regards to knowledge and behaviour regarding antibiotics. This point is supported by previous evidence that exposure to health information websites can improve knowledge about antibiotic use and AMR [39] and that offline health behaviours are liable to change because of online information [14–17]. The findings of this study, which suggest that the Internet is a viable media for the dissemination of quality information to improve behaviour with antibiotics, reinforce recommendations that health professionals should be trained to use online services such as social media to improve the dissemination of information to patients that may exhibit confusion or share misinformation through online channels [40], and that publicity campaigns should harness the public’s willingness to discuss AMR, again for example on online social media platforms, in order to produce sustained and informative dialogue [20–21].

As it is an increasingly prevalent source of health information, use of the Internet for health-related information should be considered both as a control variable in future studies of antibiotic knowledge, attitude, and behaviour, and be explored as a substantive area of interest relating to the same. Further research should interrogate 1) which Internet-based information sources in the UK, and internationally, have a positive or negative association with knowledge regarding antibiotics and, crucially, 2) whether these effects can have a consistently positive or negative impact on responsible antibiotic consumption. Following such research, interventions could be tailored to maximise the dissemination of clear and effective messaging about antibiotics and their consumption to increase public awareness, complementing other sources such as television, radio, and health professionals. One such intervention could be an increased and targeted use of information prescriptions in both human and veterinary medical settings [41] given the evidence of public willingness to use online sources of health-related information.

Finally, this study also presents consistent positive associations between education levels and better knowledge about antibiotics and issues around antibiotic use, converging with literature that suggests a positive association between education levels and correct knowledge [6, 11–12, 32–33, 42–44]. Whilst increasing levels of education did not always demonstrate larger effects than preceding levels, university levels of education did have larger effect sizes than non-university levels of education. Associations between age and sex, and knowledge about antibiotics, were less conclusive in this analysis and the analysis of the datasets in this study does not clearly support trends in either area. Conversely, in both behaviour/attitude models there were positive associations presented between some age groups and the response variables but none for education. Older respondents were more likely to report more responsible behaviours and attitudes, contradicting previous literature that suggests that older age is associated with less responsible consumption or attitudes [12, 37], supporting the suggestion that older age is associated particularly with better attitude towards consumption [43, 45–46].

### Limitations and strengths

Interpretation of these findings should consider some limitations of this study. Firstly, the datasets used were both cross-sectional in design which limits the discussion of causal links between response and predictors in the models used. Secondly, as with any survey there is the possibility of the reporting of socially desirable behaviours by participants unwilling to report their own socially undesirable behaviours. The anonymization of the individual data in both datasets is a mitigating factor for such bias.

A strength of this analysis is the use of two comparable but not identical datasets, with differing constructions of knowledge about antibiotics producing similar findings regarding the association between education levels and knowledge about antibiotics. A second strength is the use of different angles on the role of the Internet for health information, suggesting that the use of and trust in the Internet for health-related information is positively associated in the UK with knowledge about antibiotics and antibiotic resistance.

## Conclusion

The positive independent associations found in both datasets between Internet variables and both knowledge and behaviour/attitude suggest that people in the UK who use the Internet for health-related information are more likely to be better informed about and be more responsible with antibiotic medication than people that do not. The plurality of avenues for information dissemination provided by the Internet should be capitalised on by healthcare providers in both human and veterinary medical settings, for example through the use of information prescriptions for patients that make routine use of the Internet in order to guide them to consistently high-quality sources of information.
